# Understanding the Link Between Allergy and Neurodevelopmental Disorders: A Current Review of Factors and Mechanisms

**DOI:** 10.3389/fneur.2020.603571

**Published:** 2021-02-15

**Authors:** Regena Xin Yi Chua, Michelle Jia Yu Tay, Delicia Shu Qin Ooi, Kewin Tien Ho Siah, Elizabeth Huiwen Tham, Lynette Pei-Chi Shek, Michael J. Meaney, Birit F. P. Broekman, Evelyn Xiu Ling Loo

**Affiliations:** ^1^Department of Pediatrics, Yong Loo Lin School of Medicine, National University of Singapore, Singapore, Singapore; ^2^Khoo Teck Puat-National University Children's Medical Institute, National University Hospital, National University Health System, Singapore, Singapore; ^3^Department of Medicine, Yong Loo Lin School of Medicine, National University of Singapore, Singapore, Singapore; ^4^Division of Gastroenterology & Hepatology, University Medicine Cluster, National University Hospital, Singapore, Singapore; ^5^Singapore Institute for Clinical Sciences (SICS), Agency for Science, Technology and Research (A^*^STAR), Singapore, Singapore; ^6^Ludmer Centre for Neuroinformatics and Mental Health and Department of Psychiatry, McGill University, Montréal, QC, Canada; ^7^Department of Psychiatry, Onze Lieve Vrouwe Gasthuis and Amsterdam University Medical Centre, VU University Medical Center, Amsterdam, Netherlands

**Keywords:** allergic disease, neurodevelopmental disorder, mechanisms, attention deficit and hyperactivity disorder, autism spectral disorder

## Abstract

Both allergic diseases and neurodevelopmental disorders are non-communicable diseases (NCDs) that not only impact on the quality of life and but also result in substantial economic burden. Immune dysregulation and inflammation are typical hallmarks in both allergic and neurodevelopmental disorders, suggesting converging pathophysiology. Epidemiological studies provided convincing evidence for the link between allergy and neurodevelopmental diseases such as attention-deficit hyperactivity disorder (ADHD) and autism spectrum disorder (ASD). Possible factors influencing the development of these disorders include maternal depression and anxiety, gestational diabetes mellitus, maternal allergic status, diet, exposure to environmental pollutants, microbiome dysbiosis, and sleep disturbances that occur early in life. Moreover, apart from inflammation, epigenetics, gene expression, and mitochondrial dysfunction have emerged as possible underlying mechanisms in the pathogenesis of these conditions. The exploration and understanding of these shared factors and possible mechanisms may enable us to elucidate the link in the comorbidity.

## Introduction

Both allergic diseases and neurodevelopmental disorders are non-communicable diseases (NCDs) that pose a serious economic burden and impact quality of life (1–4). Allergic diseases typically start early in childhood, with atopic dermatitis (AD) being one of the earliest to manifest in the first few years of life, followed by allergic rhinitis (AR) and asthma (5). Neurodevelopmental disorders have a more variable age of onset but are usually diagnosed before the age of 7 for attention-deficit hyperactivity disorder (ADHD) (6) and diagnosed between the ages of 3 and 5 for autism spectrum disorder (ASD) (7, 8). There are postulated links between allergy and neurodevelopmental diseases (9–11). Lin et al. found that patients with ADHD and ASD had greater risks of having comorbid allergic diseases including AD, asthma, and AR (11).

**Figure 1:**
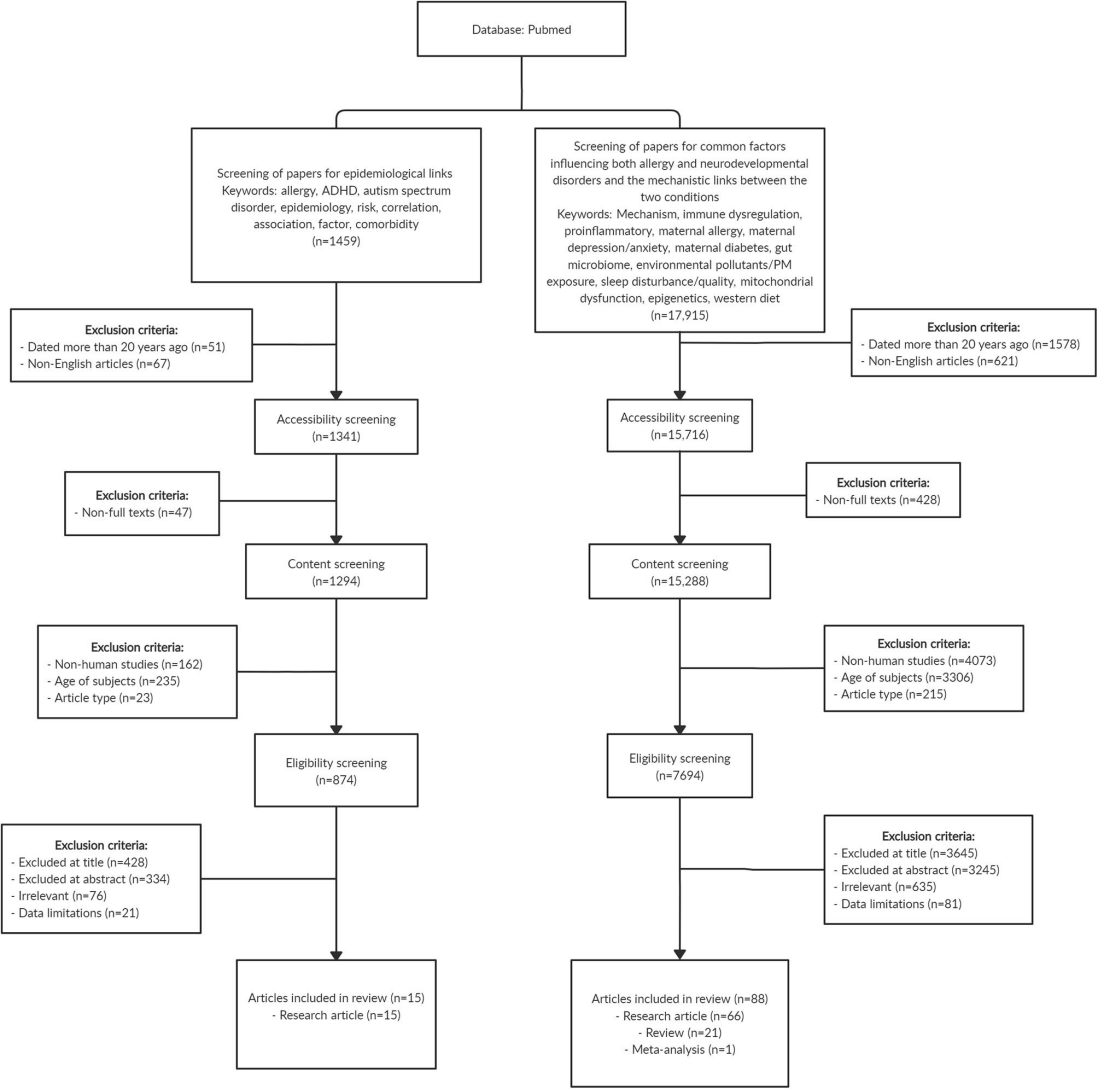
Flowchart showing the search strategy and article selection for the papers included in this review. In summary, we included 15 original research articles that reported the epidemiological link between allergy and neurodevelopmental disorders, and a total of 88 articles comprising original research (n = 66), review (n = 21), and meta-analysis (n = 1) papers that examined common factors influencing both allergy and neurodevelopmental disorders as well as the mechanistic links between the two conditions. Some of the exclusion criteria for selection of papers include age of subjects (studies that involved research subjects above 18 years old) and article type (bibliographies, biographies, legal cases, congress, editorials, interviews, letters, news articles, personal narratives, guidelines, historical articles, and retracted publications). Articles with title, abstract, and content that are irrelevant to research topic, and articles with limited data on the research topic were also excluded.

Immune dysregulation and inflammation have been well-documented as typical hallmarks in both allergic and neurodevelopmental conditions. Activation of basophils, mast cells, and eosinophils in allergic diseases release pro-inflammatory factors and cytokines (12). Elevated levels of inflammatory cytokines were similarly found to be associated with the development of ADHD and ASD (13, 14), together with upregulation of Th2 and Th17 cells in ASD (15, 16), suggesting close relations in the pathophysiology of allergy and neurodevelopmental diseases. Recent literature on the role of the immune system in various mental functions also suggests the permeation of peripheral immune cells such as macrophages across the blood–brain barrier, which alter neural functions, potentially playing a role in the development of psychiatric disorders (17). In this review, we present epidemiological evidence linking allergic diseases and neurodevelopmental disorders such as ADHD and ASD, evaluate the shared factors of allergic diseases and altered neurodevelopment, and discuss potential mechanisms underlying the comorbidity. We performed a literature search on PubMed database and identified original research, review, and meta-analysis articles that are relevant to the research topic. Figure 1 shows the literature search strategy for articles that are included in this review.

## Epidemiological Link between Allergy and Attention-Deficit Hyperactivity Disorder

ADHD is one of the most common childhood neurodevelopmental disorders (18). According to *Diagnostic and Statistical Manual of Mental Disorders* (Fifth Edition) (DSM-5) criteria, a child is diagnosed with ADHD if he presents six or more symptoms of inattention, hyperactivity and impulsivity, or both for a period of at least 6 months that has affected daily activities and functioning appropriate for age (19).

A systematic review and meta-analysis provided strong evidence for the link between allergy and ADHD; patients with allergy have a 30–50% greater risk of developing ADHD (20). Evidence for the association between allergic diseases and ADHD in children is also presented in a number of longitudinal studies. A study by Genuneit et al. comprising a birth cohort of 770 children in Germany reported a significant association between early AD in the first 4 years of life and ADHD development by 8 years of age (21). In the Twin study of CHild and Adolescent Development (TCHAD), children who had asthma at 8–9 years old had nearly double the risk of having hyperactivity and impulsivity symptoms at 13–14 years old than those without asthma, even after adjusting for previous ADHD symptoms (22). Additionally, when analyzing data from each pair of twins, the authors found that 68% of the phenotypic associations between asthma and hyperactivity–impulsivity symptoms originated from genetic sources, underscoring the potential role of genetics in linking allergy and neurodevelopmental disorders (22). In another longitudinal study in Taiwan comprising 18,473 toddlers aged 1 month up to 3 years with AD, the authors found a significant relationship between AD and ADHD as compared with healthy controls without AD (23). Concomitant AR, asthma, and allergic conjunctivitis further increased the odds ratio of developing ADHD than in those with only AD (23).

Besides cohort studies, cross-sectional studies also present data in support of the association between allergy and ADHD. Strom et al. analyzed 19 population-wide surveys in the United States and reported that both AD and asthma were significantly correlated with increased risks for ADHD in childhood (24). In a cross-sectional study of 2,772 children aged 3–6 years in Taiwan, Yang et al. reported a significant relationship between AD with allergen sensitization and ADHD (OR: 4.50, 95% CI: 1.28–15.86), as well as between asthma with allergen sensitization and ADHD (OR: 3.65, 95% CI: 1.07–12.49) (25). Similarly, a large population-based case–control study conducted in Taiwan showed that children with AD and asthma had a 1.48 times increased risk of developing ADHD (26). The relative risk of asthma and AR was 1.60 and 1.38 times higher, respectively, in a cohort of 549 Korean children with ADHD than in controls without ADHD (27). Jiang et al. conducted a cross-sectional study in China comprising school-age children from 5 to 12 years old and found food allergy to be significantly associated with ADHD (28). In addition, the risk of ADHD was elevated with increased number of allergic conditions (28). Taken together, current evidence in the field suggests that there is a strong association between allergy and ADHD and that children with allergy may be at increased risk of developing ADHD. Table 1 presents the results from studies on allergy and ADHD.

**Table 1:** Summary of epidemiological link between allergy and ADHD.

**Title of article**	**Country**	**Study design**	**Sample size**	**Age range in years**	**Allergic conditions**	**Outcome**
Infant atopic eczema and subsequent attention-deficit hyperactivity disorder – A prospective birth cohort study (21)	Germany	Prospective birth cohort study	770	Birth to 11	•Atopic eczema (AE)	•Parental reported AE in early life up to 4 years was significantly associated with both early and late ADHD (RR: 1.92, 95% CI: 1.03–3.57) •Doctor diagnosed AE in first 4 years of life was significantly associated with early development of ADHD in first 8 years of life (RR: 2.58, 95% CI: 1.11–5.99)
Association between childhood asthma and ADHD symptoms in adolescence—A prospective population-based twin study (22)	Sweden	Prospective population-based twin study	1,812	First timepoint: 8–9 Second timepoint: 13–14	•Asthma	•Asthma at 8–9 years was significantly linked with elevated risks of 4 (OR: 2.77, 95% CI: 1.23–6.26), 5 (OR: 3.62, 95% CI: 1.04–12.57), or 6 (OR: 8.36, 95% CI: 1.52–45.81) symptoms of inattention at 13–14 years •Children with asthma at 8–9 years exhibited more than double the odds of having 3 hyperactivity–impulsivity symptoms at 13–14 years (OR: 2.50, 95% CI: 1.46–4.28)
Longitudinal association between early atopic dermatitis and subsequent attention-deficit or autistic disorder (23)	Taiwan	Population-based case–control study	18,473 AD, 18,473 controls	1 month−3 years	•AD •Asthma •AR •Allergic conjunctivitis	•Children with AD had significantly earlier age of diagnosis for ADHD compared with controls (*p* < 0.001) •AD in children was significantly associated with elevated risks of development of ADHD (HR: 2.92, 95% CI: 2.48–3.45) and ASD (HR: 8.90, 95% CI: 4.98–15.92) •Comorbid asthma, AR, and allergic conjunctivitis further increased the risk of ADHD (HR: 4.67, 95% CI: 3.81–5.43) and ASD (HR: 16.25, 95% CI: 8.63–30.60)
Association between atopic dermatitis and attention deficit hyperactivity disorder in U.S. children and adults (24)	United States	Cross-sectional population-based surveys	354,416 children 34,613 adults	Children: 2–17 Adults: 18 and above	•AD •Asthma •Hay fever	•AD in children was significantly linked with elevated risks of ADHD (*p* < 0.05) after adjustment for health-care utilization, sociodemographic data, and comorbid allergic disease •AD in adults was significantly linked with elevated risks of ADHD (*p* < 0.05) after adjustment for health-care utilization, sociodemographic data, and comorbid allergic disease •All severity levels of ADHD including mild, moderate, and severe were significantly linked with elevated risks of AD (*p* < 0.05)
Association between allergic diseases, allergic sensitization and attention-deficit/hyperactivity disorder in children: A large-scale, population-based study (25)	Taiwan	Population-based cross-sectional survey	2,772	3–6	•AD •Asthma •Allergic rhinitis •Allergic conjunctivitis •Food allergy	•AD comprising allergic sensitization was significantly linked with ADHD (*p* < 0.05) after adjustment for age, birth weight, sex, maternal history of allergy, maternal education, and period of breastfeeding •Asthma comprising allergic sensitization was significantly linked with ADHD (*p* < 0.05) after adjustment for age, birth weight, sex, maternal history of allergy, maternal education, and period of breastfeeding •Sensitization to mite allergen was significantly associated with ADHD (*p* < 0.05)
Association between atopic diseases and attention-deficit hyperactivity disorder in childhood: A population-based case-control study (26)	Taiwan	Population-based case–control study	4,692 ADHD, 18,768 controls	Up to 18	•AD •Asthma •Allergic rhinitis •Allergic conjunctivitis	•AD, AR, asthma, and allergic conjunctivitis were all significantly linked with ADHD independently (*p* < 0.001) •Increasing number of allergic diseases was significantly associated with increasing risk of ADHD after adjustment for age, sex, and urbanization (*p* < 0.001)
The associations between ADHD and asthma in Korean children (27)	Korea	Longitudinal study	549 ADHD, 3,564 controls	7–8	•AD •Asthma •AR •Allergic conjunctivitis •Food allergy •Drug allergy	•Significantly higher proportion of children in the ADHD group had a history of asthma (*p* < 0.001) and AR (*p* < 0.001) compared with those in the control group •Compared with controls, a significantly higher proportion of children in the ADHD group had received treatment for asthma in the past 12 months (*p* = 0.031) •Compared with controls, a significantly higher proportion of children in the ADHD group had received treatment for AR in the past 12 months (*p* = 0.007)
Early food allergy and respiratory allergy symptoms and attention-deficit/hyperactivity disorder in Chinese children: A cross-sectional study (28)	China	Cross-sectional study	22,018	Mean age: 8.8 ± 1.8	•Food allergy •AR •Asthma	•Food allergy was significantly associated with elevated risks of ADHD (*p* < 0.001) •Food allergy with concurrent AR and/or asthma further increased the risk of ADHD (*p* < 0.001)

*AE, atopic eczema; AD, atopic dermatitis; AR, allergic rhinitis; ADHD, attention-deficit hyperactivity disorder; RR, relative risk; OR, odds ratio; HR, hazard ratio*.

## Epidemiological Link Between Allergy and Autism Spectrum Disorder

ASD, commonly known by the term autism, is a condition that causes impairment in a wide range of cognitive–emotional and social functions of affected individuals (29). Diagnosis is commonly conducted with the use of the DSM-5 (19), which names the following as common symptoms for diagnosis: (1) persistent impaired social skills, (2) limited and repetitive behavior with the need to follow rigid routines, and (3) heightened or dull reactions to sensory stimuli. Apart from developmental and behavioral problems (30), individuals with ASD have been commonly found to suffer from other comorbidities such as allergic diseases.

Evidence from a number of longitudinal studies showed that subjects who developed allergic conditions such as AD and asthma in early life had an increased risk of being diagnosed with ASD subsequently. A Taiwanese study found that children who developed allergic conditions before the age of 3 had increased risk of being diagnosed with ADHD and ASD; and interestingly, this risk increased with the number of allergic comorbidities (31). Another study conducted in Taiwan also reported a correlation between AD diagnosis before age 2 and increased risk of ADHD and ASD. This risk increased even further in children with severe AD and with concomitant atopic respiratory diseases (32). Additionally, children who developed asthma before the age of 3 also had a higher cumulative incidence and risk of being diagnosed with ASD at the 8-year follow-up (33).

Supporting evidence was also provided by cross-sectional studies. A recent study by Xu et al. observed significant positive relationships between ASD and skin, respiratory, and food allergy among children in the United States, with food allergy showing the strongest association (34). In another study conducted in the United States, children with lower Ages & Stages Questionnaire (ASQ) communication scores at age 3 had up to three times increased risk of being diagnosed with food allergy, while children with AD at age 3 had approximately double the risk of having a low ASQ communication score (35). The positive relationship between food allergy and ASD is further supported by results from the CHildhood Autism Risk from Genetics and the Environment (CHARGE) study, which compared the allergy profile of 560 children with ASD and 391 non-ASD children and noted a higher prevalence of food allergy in the group of children with ASD (36). While most studies point to the positive association between allergy and ASD, a study conducted on 30 children with ASD in Turkey found no associations between ASD and allergic symptoms (37). This could be due to the small sample size involved, limiting statistical power. However, a larger cross-sectional study in the United States comprising 77,951 children provided evidence for a positive correlation between ASD and allergy, where the prevalence of asthma was 35% higher in children with ASD (38). Table 2 presents the results of studies on allergic diseases and ASD.

**Table 2:** Summary of epidemiological link between allergy and ASD.

**Title of article**	**Country**	**Study design**	**Sample size**	**Age range in years**	**Allergic conditions**	**Outcome**
Is atopy in early childhood a risk factor for ADHD and ASD? A longitudinal study (31)	Taiwan	Longitudinal follow-up study	14,812 atopic, 6,944 non-atopic	10–13 at end of follow-up	•Asthma •AR •Allergic conjunctivitis •AD	•Higher proportion of children with atopic disorders in early life developed ASD in later life (0.8 vs. 0.2%, *p* < 0.001) compared with children without atopic disorders •Higher proportion of children with atopic disorders in early life developed ADHD (6.3 vs. 2.9%, *p* < 0.001) in later life compared with children without atopic disorders •Increase in atopic comorbidities increased the risk of ASD development in children (HR: 4.29, 95% CI: 2.25–8.19 for children with four atopic conditions compared with HR: 2.14, 95% CI: 0.90–5.11 for children with only 1 atopic condition)
Comorbidity of atopic disorders with autism spectrum disorder and attention deficit/hyperactivity disorder (32)	Taiwan	Population-based longitudinal cohort study	387,262 with AD, 387,262 without AD	6–10 at end of follow-up	•AD •AR •Asthma	•AD diagnosis before 2 years of age was associated with higher risk of ASD (HR: 1.10; 95% CI: 1.03–1.18) and ADHD (HR: 1.16; 95% CI: 1.13–1.19) development •Early onset of respiratory diseases before 2 years of age was associated with higher risk of ASD (HR: 1.49; 95% CI: 1.35–1.64) and ADHD (HR: 1.68; 95% CI: 1.62–1.75) development
Increased risk of autism spectrum disorder among early life asthma patients: an 8-year nationwide population-based prospective study (33)	Taiwan	Population-based prospective study	2,134 asthmatic infants and children, 8,563 controls	8–11 at end of follow-up	•Asthma	•Higher proportion of children with asthma in early life developed ASD in later childhood (1.3% vs. 0.7%, p = 0.007) compared with children without asthma. •After adjustment for age, gender, urbanization level, and allergy comorbidities, infants and children with asthma had an increased risk of ASD development in later life (HR: 2.01, 95% CI: 1.19–3.40) •Asthmatic subjects who later developed ASD entered asthma remission at an older age as compared with non-ASD asthmatic subjects (5.41 ± 3.09 vs. 4.30 ± 3.04 years, *p* = 0.059) •Asthmatic subjects who later developed ASD experienced an increased duration of asthma (3.83 ± 3.25 vs. 2.97 ± 2.94 years, *p* = 0.132) as compared with those without ASD
Association of food allergy and other allergic conditions with autism spectrum disorder in children (34)	United States	Cross-sectional population-based surveys	199,520 children	3–17	•Food allergy •Respiratory allergy •AD or other skin allergy	•Increase in weighted prevalence of reported food, respiratory, and skin allergies in children with ASD (11.25, 18.73, and 16.81%, respectively), as compared with children without ASD (4.25, 12.08, and 9.84%, respectively) •Significantly positive association between food allergy (OR: 2.29; 95% CI: 1.87–2.81), respiratory allergy (OR: 1.28; 95% CI: 1.10–1.50), and skin allergy (OR: 1.50; 95% CI: 1.28–1.77) and ASD after adjusting for sociodemographic data and other allergies •OR of ASD was 1.82, 95% CI: 1.62–2.04 (*p* < 0.001) in children with allergy as compared with those without
Allergic disease and low ASQ communication score in children (35)	United States	Cross-sectional population-based surveys	715 children	Birth to 8.5 ASQ score taken at age 3; food allergy reported at ages 3 and 6	•AD •Food allergy •Asthma	•Higher proportion of children with lower ASQ score were diagnosed with ASD by age 8 compared with those with normal scores (11.1 vs. 1.2%; *p* < 0.001), •Higher proportions of children with lower ASQ score reported having food allergy at age 6 (17.6% for low ASQ score group vs. 4.7% for normal development group; *p* = 0.007) •Children with food allergy at ages 3 or 6 were approximately three times more likely to have lower ASQ scores (*p* = 0.030) •Children with AD diagnosis at age 3 were twice as likely to have a low ASQ score (*p* = 0.054) as compared with those without AD
Asthma and allergies in children with autism spectrum disorders: results from the CHARGE Study (36)	United States	Population-based case–control study	560 ASD, 391 controls	2–5 at study enrolment	•Asthma •Food allergy	•Risk of asthma did not differ in ASD and non-ASD subjects (16%, *p* = 0.93) •Children with ASD were two times as likely to report having food allergies (Adj OR: 2.23, 95% CI: 1.28–3.89)
Co-occurrence of autism and asthma in a nationally representative sample of children in the United States (38)	United States	Cross-sectional population-based surveys	77,951 children	3–18	•Asthma	•Risk of asthma is higher in children with ASD after adjustment for sociodemographic factors, body mass index, prior brain injury, and exposure to passive smoke (Adj OR: 1.19, 95% CI: 1.03–1.36)

*AD, atopic dermatitis; AR, allergic rhinitis; ADHD, attention-deficit hyperactivity disorder; Adj OR, adjusted OR; ASD, autism spectrum disorder; ASQ, Ages & Stages Questionnaire; HR, hazard ratio; OR, odds ratio*.

## Common Factors Implicated in Both Allergic Diseases and Neurodevelopmental Disorders

Evidence from a number of studies had highlighted common factors such as maternal depression, anxiety, gestational diabetes, maternal allergic status, environmental factors, microbiome dysbiosis, and sleep disturbances in both neurodevelopmental and allergic diseases. In this next section, we present evidence on the involvement of these factors in neurodevelopmental and allergic diseases.

### Maternal Depression and Anxiety

The link between allergy and neurodevelopmental disorders may be established before birth, during the critical preconception and prenatal period. There is increasing evidence to show that stress, depression, or anxiety in the mother before or during pregnancy and after birth can have far-reaching effects on the infant, rendering them more susceptible to development of allergic diseases (39, 40) and/or neurodevelopmental disorders (41, 42). Maternal depression leads to changes in glucocorticoid sensitivity and an imbalance in pro-inflammatory cytokines levels (43, 44), potentially altering the immunological profile of the offspring. In addition, the decrease in placental monoamine oxidase A in depression causes elevated levels of serotonin (45) that may negatively impact fetal brain development (46).

In the Born in Guangzhou Cohort Study (BIGCS), Wei et al. studied 8,580 mother–infant pairs to investigate the link between maternal depressive symptoms during pregnancy and subsequent AD development in the infant at 12 months (47). They found maternal depressive symptom scores to be positively associated with the risk of AD in the infant during the 1st year of life (47). Similarly, in another study of two birth cohorts including the Cohort for Childhood Origin of Asthma and Allergic Diseases (COCOA) cohort and the Panel Study on Korean Children (PSKC) cohort, prenatal maternal anxiety and depression were found to be significantly associated with elevated risks of AD in the infant in both cohorts (48).

Analogous associations have been reported in studies involving ADHD subjects. Van der Bergh and Marcoen found prenatal maternal anxiety to be significantly associated with ADHD traits at 8 and 9 years old (49). Elevated serum IgE levels were also reported in children of mothers who experienced prenatal distress, another indicator of Th2-biased immune responses (48). Maternal levels of pro-inflammatory IL-6 and C-reactive protein levels in the third trimester were associated with offspring cognitive functioning and behavior (50).

### Gestational Diabetes Mellitus

Gestational diabetes mellitus (GDM) has been reported to be a risk factor for both allergic diseases and ASD. A positive association was found between GDM and development of asthma and wheezing in the offspring (51, 52). The risk of early childhood AD was reportedly increased by 7.5 times and allergen sensitization by six times in infants born to mothers with GDM (53). Similarly, a meta-analysis also revealed the risk of ASD in offspring to be twice as high in mothers with GDM (54). Furthermore, in a cohort of 419,425 children in the United States, the risk of ASD development in the offspring was increased when the mother had type 1 diabetes, type 2 diabetes, and GDM (55). This may be due to the induction of a pro-inflammatory state through the increase in levels of cytokines including TNF-α and IL-6 (56) and the decrease in anti-inflammatory factors such as IL-10 in GDM (57). This inflammatory profile was also observed in ADHD patients where serum IL-6 levels were significantly higher than in healthy controls (58), while TNF-α levels had been reported to be elevated in children with ASD (59). Besides this, the severity of ASD (60) and AD (61) had been reported to increase with the levels of pro-inflammatory cytokines.

### Maternal Allergic Status

The influence of maternal allergy on the development of allergic diseases in the offspring is well-documented. An analysis in the PRogramming of Intergenerational Stress Mechanisms (PRISM) cohort comprising 553 mother–child pairs showed that offspring born to mothers with allergic history had elevated risks of developing asthma (62). Another study also reported higher risk of AD and AR development in offspring born to mothers with history of asthma and AR during pregnancy, respectively (63). Maternal history of asthma was significantly correlated with greater risks of wheezing in the child (64). Likewise, maternal asthma has been reported to increase the risk of ADHD (65) development, while maternal asthma and allergy were found to increase the risk for ASD in offspring (66). Possible mechanisms suggested for these associations include epigenetic influences (67), exposure to common environmental conditions (68), and alterations to immunological profiles (66). Interestingly, it is noteworthy that the effects of maternal allergy on offspring outcomes may follow sex-specific patterns. Clifton et al. highlighted that female infants of asthmatic mothers who did not use steroid inhalers had significantly lower birth weights, but male infants were not affected (69). The sex-specific effect of maternal allergy on birth outcomes was also observed in the association between maternal atopic status and risk of ADHD in offspring. Cowell et al. analyzed a cohort comprising 250 mother and child pairs and found that maternal atopic status was associated with greater risk of ADHD behavior in the child, and this association was more prominent for female infants (70).

### Environmental Factors

#### Diet

Environmental factors such as diet may play a role in influencing the development of both allergy and neurodevelopmental disorders. In a review by Skypala et al. the authors presented compelling evidence on how a western diet is composed of refined grains, high fat, and sugar was significantly correlated with wheezing in children, and countries with westernized lifestyles were especially at risk of higher food allergy rates (71). Julia et al. highlighted the increase in prevalence of allergic diseases with westernized lifestyles and diet, and they proposed mechanisms by which saturated fatty acids lead to allergic inflammation and how a lack of fiber and several vitamins in western diets result in the loss of protective effects against systemic inflammation (72). Similarly, ADHD patients were observed to have increased intake of refined grains and lower intake of vitamin B2 and dairy than have controls (73). In the Raine study, a western diet high in refined sugars and fat has been found to be linked with ADHD (74). Adverse impact of western diet on hippocampal functioning and neuroinflammation has been reported in a review by Noble et al. (75). Moreover, dietary differences may contribute to gut microbiome dysbiosis observed in patients with allergy and neurodevelopmental disorders (75, 76).

#### Exposure to Environmental Pollutants

Early life exposure to particulate matter (PM) or other pollutants may influence the development of both allergy and neurodevelopmental disorders. There is strong evidence to support the link between environmental PM and allergic disorders, as demonstrated in various studies (77–80). A review by Wu et al. highlighted the detrimental impact of PM exposure on asthma and AR and discussed the possible mechanisms by which different PM subtypes could elicit downstream inflammation and oxidative stress (77). Another prospective birth cohort study of 2,860 children in Munich also found a significant relationship between PM_2.5_ exposure and asthma as well as between NO_2_ exposure and eczema (78). Exposure to toxic metals has been associated with the development of allergic disease. In a study by Kim et al. involving 4,350 children aged 7–8 years, the authors found mercury exposure to be correlated with risk of asthma (81). Mercury levels in the blood were also found to be positively correlated with wheezing outcomes and airway hyperresponsiveness (81). In the Mothers and Children's Environmental Health (MOCEH) study comprising a cohort of 1,061 mother–infant pairs, the authors highlighted mercury exposure at 2 years to be a risk factor for AD development in later childhood (82).

The link between PM exposure and ADHD was similarly discussed in a review by Myhre et al. (83). Prenatal and postnatal exposure to PM was found to be correlated with increased risk of displaying behavioral problems and ADHD symptoms. Additionally, exposure to toxic metals present in PM was also positively associated with ADHD behavior (83). In another study of 116 children aged 8 years or younger diagnosed with ADHD, having living quarters close to streets or motorways resulting in higher exposure to traffic PM was associated with higher risk of developing ADHD (84). Other forms of environmental pollutants including toxic metals such as mercury and lead had also been postulated as potential risk factors for ASD (85). Likewise, a study of 284 children with ASD found a possible link between high concentration of toxic metals in ambient air with ASD (86).

### Microbiome Dysbiosis

The microbiome is another key player in the link between allergy and neurodevelopmental disorders. It is well-established that gut microbiome dysbiosis plays an important role in allergic diseases such as AD (87), and similar shifts in host microbiome are increasingly being elucidated in ADHD patients (88). Additionally, the role of microbiome in influencing allergy and neurodevelopmental disorders may stem from maternal factors such as stress during the prenatal period, as well as differing dietary patterns. Zijlmans et al. studied a cohort of 56 infants and found that maternal stress was linked with dysregulation of the infant intestinal microbiome, characterized by elevated levels of pathogenic species such as *Enterobacter* and decreased levels of commensal *Bifidobacteria* and *Lactobacillus*, contributing to an overall increase in inflammatory state (89). These same bacterial species have also been implicated in neurodevelopmental disorders. A review conducted by Bundgaard-Nielsen et al. (90) on the gut microbiota profiles of ADHD and ASD patients presented convincing evidence on altered gut bacterial composition in ASD individuals as compared with healthy controls, which included increase in *Bacteroides* and *Clostridium* as well as the decrease in *Bifidobacterium, Streptococcus*, and *Prevotella* in stool microbiota. On the other hand, abundance of *Parabacteroides, Prevotella*, and *Lactobacillus* were decreased in ADHD patients. A prospective study on 93 children from infancy to 8 years of age reported decreased *Bacteroides, Prevotella*, and *Coprococcus* in stool samples of children with allergy as compared with healthy children across various time points, while *Lactobacillus, Enterococcus*, and *Lachnospira* were found to be decreased and *Bifidobacterium* increased in children with allergy at 8 years of age (91). Another study on 89 children from the United States found that gut microbiome at age 1 affects cognitive function at age 2. Children with abundance of *Bacteroides* and *Faecalibacterium* were found to exhibit the best and worst cognitive capabilities, respectively (92).

Szopinska-Tokov et al. found that ADHD patients had lower beta diversity in their gut microbiome than controls (93). Additionally, a specific genus, *Ruminococcaceae_UCG_004*, was linked to symptoms of inattention (93). In another review by Cenit et al. the authors discuss how specific bacteria in the gut have the capability to produce and influence important neurotransmitters including gamma-aminobutyric acid (GABA) and serotonin (88). Downregulation of *Bifidobacterium* and *Lactobacillus*, which produce GABA, as well as *Streptococcus* spp. and *Enterococcus* spp., which produce serotonin in the stool microbiota of ASD and ADHD patients have been observed (90). Serotonin and GABA produced by gut bacteria can potentially interact with epithelial cells to stimulate the release of other cytokines and hormones, which in turn regulate neuronal signaling to influence brain functioning (88). Higher abundance of Neisseriaceae and Bacteroidaceae and a significant reduction in gut microbiome diversity was also observed in the ADHD group as compared with controls (94). Similar to these findings, increased abundance of Bacteroidaceae in the gut has previously been found to be associated with AD groups compared with controls (95, 96).

### Sleep Disturbances

Many studies have reported that AD disrupts sleep quality, especially so when AD is coupled with other comorbidities such as AR and asthma (97–100). Disease severity of AD was found to be inversely correlated with sleep quality, with scratching being one of the main reasons for poor sleep (98). A study in Korea reported increased sleep problems among children with AR and AD. The severity of the sleep problems was positively correlated to the percentage of eosinophils measured in the peripheral blood (101). Inadequate sleep, when coupled with AD, resulted in a 2-fold increase in the risk of ADHD compared with that in subjects with AD and adequate sleep (24). These findings were replicated in another study of 6,484 children based in Germany, whereby the risk of ADHD was significantly higher in children with both atopic AD and sleep disturbances (102). Disturbances to sleep can lead to abnormal functioning of various brain circuits, including the prefrontal cortex, which in turn influences behavior and cognitive operations (103). Executive functioning is usually defective in ADHD, and hence, poor sleep quality may collectively enhance the probability of developing symptoms of ADHD in patients with allergic diseases (104). Similarly, a study by Mazurek et al. highlighted a significant link between behavioral problems in ASD children and poor sleep (105). Sleep disturbances are known to aggravate various behavioral problems in ASD patients and disrupt daytime functioning (106).

## Possible Mechanisms Linking Allergy and Neurodevelopmental Disorders

Apart from inflammation, which is the common underlying mechanism for allergy and neurodevelopmental disorders, epigenetics and gene expression and mitochondrial dysfunction have emerged as possible mechanisms linking the two conditions.

### Epigenetics and Gene Expression

Environmental exposure in early life is linked to the development of allergic and neurodevelopmental diseases (107, 108), and epigenetic mechanisms, which conveyed genomic adaptation to environmental factors, may explain the effects of the environment on the development of allergic and neurodevelopmental conditions (109, 110).

Xu et al. conducted a large-scale epigenome-wide association study in the whole blood of 392 children with asthma and 1,156 controls aged 4–8 years, and they found 14 differentially methylated CpG sites that were subsequently validated through meta-analysis of six independent European cohorts (111). The methylated CpG sites were associated with whole blood transcriptional profiles related to increased activation of eosinophils, cytotoxic T cells, and natural killer cells and reduced number of naive T cells (111). Similarly, another epigenome-wide meta-analysis study also reported CpG methylation and differentially methylated regions in children with asthma, and pathway analyses showed that the methylated CpG and differentially methylated regions were associated with asthma-related immune processes (112).

van Mil et al. assessed the DNA methylation profiles of selected genes that are involved in neurotransmitter system and neurodevelopment in the cord blood samples of an ongoing population-based birth cohort (113). The group found that lower methylation levels of seven genes assessed at birth were associated with more ADHD symptoms at 6 years of age (113). More recently, a large epigenome-wide association study of childhood ADHD reported different DNA methylation profiles and differentially methylated sites between ADHD cases and controls (114). The differentially methylated sites were associated with ADHD risk variants, suggesting that genetic risk factors may influence ADHD through epigenetic mechanism (114).

Taken together, epigenetic and gene expression may be a common underlying mechanism in the etiology of allergy and neurodevelopmental disorders. Interestingly, a study conducted in a mouse model of maternal allergic asthma provides evidence that allergy and neurodevelopmental disorders may be linked by epigenetic mechanisms (115). The offspring from female mice with allergic asthma showed behavioral abnormalities including deficits in social interactions, increased marble burying, and decreased grooming as compared with offspring from female mice without allergic asthma (115). Microglia isolated from the brains of offspring with maternal allergic asthma had differentially methylated regions enriched for immune signaling pathway and important microglial developmental transcription factor-binding motifs. Moreover, both differentially methylated regions and differentially expressed genes found in microglia of offspring with maternal allergic asthma significantly overlapped with genes that are differentially expressed in the human ASD cortex (115). These findings suggest that maternal allergic asthma can cause neurodevelopmental disorder such as ASD in offspring, and epigenetics may be the underlying mechanism linking allergy and ASD.

### Mitochondrial Dysfunction

Mitochondrial dysfunction is characterized by loss of efficiency in electron transport chain (ETC) and reduced production of energy in the form of adenosine triphosphate (ATP), which is essential for cellular metabolism and function. The malfunctioning is driven by oxidative stress and reactive oxygen species (ROS) production, and malfunctioning mitochondria alter the bioenergetics and metabolic profile of the cell to favor systemic inflammation that plays a central role in the pathogenesis of chronic diseases, e.g., allergy and neurodevelopmental disorders (116, 117).

Raby et al. reported a significant association between mitochondrial haplotype and total serum IgE levels and that haplogroup U carriers had greater skin prick test reactivity and higher rate of AD (118). A recent study by Ederle et al. showed that peripheral blood mononuclear cells (PBMCs) of severe asthmatic patients had increased mitochondrial respiratory chain complexes activity and ROS production than had PBMCs of healthy subjects (119). Mitochondrial dysfunction in the form of reduced cytochrome *c* expression and activity and decreased lung ATP levels was observed in ovalbumin (OVA)-induced experimental allergic asthma mice, and anti-inflammatory IL-4 antibody treatment was able to reverse the mitochondrial dysfunction, indicating a link between mitochondrial dysfunction and inflammation in allergic asthma (120). Moreover, induced mitochondrial dysfunction in airway epithelial cells demonstrated exacerbated antigen-driven allergic airway inflammation, suggesting a role of mitochondrial function in regulating inflammation associated with allergic conditions (121).

In a study involving eight children with ASD and eight controls aged 4–10 years, significantly lower ETC complex activities were observed in the cerebellum, frontal cortex, and temporal cortex of the ASD group (122). The reduction in ETC complex activities was also reported in other studies that examined mitochondrial dysfunction in brain of ASD patients (123, 124). Verma et al. generated cybrid cell lines by fusing platelets from ADHD and non-ADHD subjects with neuroblastoma cell line devoid of mitochondrial DNA to investigate mitochondrial pathology in ADHD (125). The group demonstrated significantly lower cellular and mitochondrial respiration, reduced ATPase transcript levels, reduced mitochondrial complex activity, loss of mitochondrial membrane potential, and elevated oxidative stress in ADHD cybrid neurons, suggesting a role of mitochondrial pathology in ADHD (125).

Collectively, mitochondrial dysfunction may bridge the gap for understanding how allergy and neurodevelopmental disorders are related (126).

## Discussion

The epidemiological link between allergy and neurodevelopmental disorders such as ADHD and ASD has been established in multiple cross-sectional and longitudinal studies (24, 25, 32, 34). While the cross-sectional studies showed a strong association between allergy and neurodevelopmental disorders (25, 28, 35, 38), the longitudinal data indicated that allergy in early life is a strong risk factor for development of ADHD and ASD in later life (27, 31, 32). Hence, the identification of this group of at-risk subjects with pre-existing allergy conditions may allow early treatment and intervention for ADHD and ASD.

We also discussed common factors that contribute to the development of both allergy and neurodevelopmental disorders, and they included maternal depression and anxiety (43–46), GDM (56, 57), maternal allergic status (65, 66), environmental factors such as diet and exposure to environmental pollutants (77, 83), microbiome dysbiosis (90, 96), and sleep disturbances (98, 102). However, there is a lack of direct evidence showing that these factors can connect both allergy and neurodevelopmental disorders. For instance, the risk of developing ADHD and ASD at later childhood for children with AD born to mothers of depression has not been investigated. Therefore, more longitudinal studies are needed to study the association between the factors and both conditions in the same cohort and also examine the mediating effect of these factors in the association between allergy and neurodevelopmental disorders.

With emerging evidence on the possible links between allergy and neurodevelopment disorders, it is important to understand the underlying mechanisms so as to identify new treatment and therapeutic targets. To further elucidate the role of epigenetics (111, 114) and mitochondrial dysfunction (119, 122) in contributing to the link and development of allergy and ADHD and ASD, more *in vivo* studies are required to examine the methylation pattern and ETC complex activities in animal models that are reflective of the conditions.

## Conclusions

**Figure 2:**
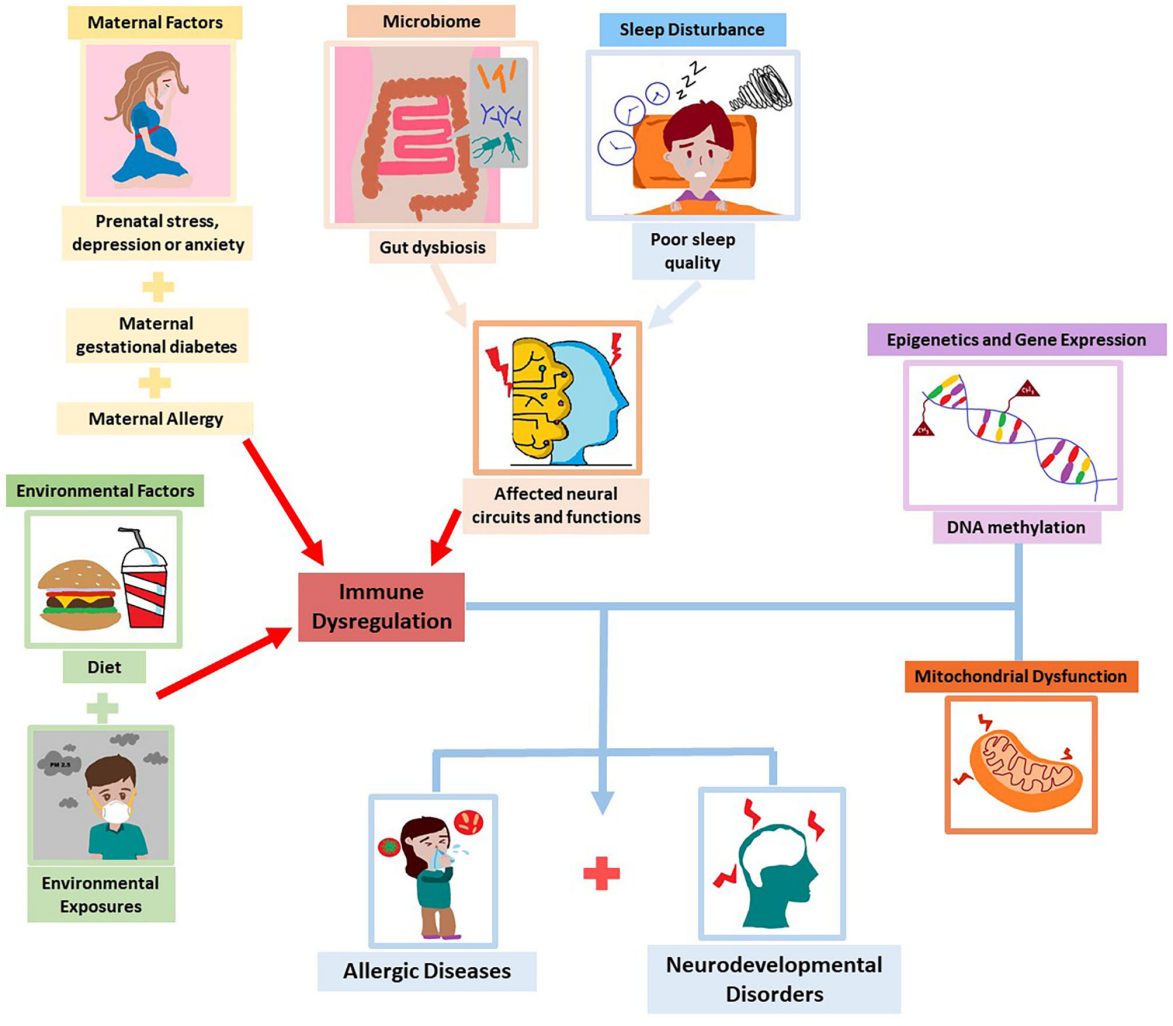
Common factors and mechanisms underlying allergic diseases and neurodevelopmental disorders.

In this narrative review, we have discussed the association between allergic diseases and neurodevelopmental disorders such as ADHD and ASD, that both start early in life. Early prevention would be of utmost importance as both disorders pose a serious burden and impact on quality of life. Recent studies reported several factors such as maternal depression and anxiety, GDM, maternal allergic status, diet, exposure to environmental pollutants, microbiome dysbiosis, and sleep disturbances that influence the development of both allergy and neurodevelopmental disorders. Moreover, apart from inflammation, epigenetics and gene expression and mitochondrial dysfunction have emerged as possible underlying mechanisms in the pathogenesis of these conditions. The exploration and understanding of these shared factors and possible mechanisms may enable us to elucidate the link in the comorbidity (Figure 2).

## Author Contributions

RC, MT, and DO performed literature search and wrote the manuscript. KS, ET, LS, and MM critically reviewed and revised the manuscript. EL conceptualized the ideas, critically reviewed, and wrote the manuscript. All authors contributed to the article and approved the submitted version.

## Conflict of Interest

The authors declare that the research was conducted in the absence of any commercial or financial relationships that could be construed as a potential conflict of interest.

## Abbreviations

AD: atopic dermatitis
AR: allergic rhinitis
ASD: autism spectrum disorder
ATP: adenosine triphosphate
ADHD: attention-deficit hyperactivity disorder
DSM: Diagnostic and Statistical Manual of Mental Disorders
ETC: electron transport chain
GDM: gestational diabetes mellitus
NCD: non-communicable disease
OVA: ovalbumin
PM: particulate matter
PBMC: peripheral blood mononuclear cells
ROS: reactive oxygen species.
